# Does COVID-19 Fear Induce Employee Innovation Performance Deficiency? Examining the Mediating Role of Psychological Stress and Moderating Role of Organizational Career Support

**DOI:** 10.3390/ijerph191610422

**Published:** 2022-08-21

**Authors:** Md Altab Hossin, Lie Chen, Md Sajjad Hosain, Isaac Owusu Asante

**Affiliations:** 1 School of Innovation and Entrepreneurship, Chengdu University, No. 2025, Chengluo Avenue, Chengdu 610106, China; 2 Business School, Sichuan University, Chengdu 610065, China; 3 School of Economics and Management, Southwest Jiaotong University, No. 111, Section 1, North Second Ring Road, Chengdu 610031, China

**Keywords:** fear of infection, COVID-19, pandemic, psychological stress, organizational career support, employee innovative performance, moderated mediation

## Abstract

With the immense, short/long-term, and multidirectional effects of the coronavirus disease (COVID-19) pandemic on work performance, industry activities, and the national/global economy, it has adversely affected employees’ psychological well-being due to its elevated stress and anxiety that have substantially affected employee innovation performance (deficiency) (EIP(D)). The goal of this empirical paper is to identify how COVID-19 induces EIPD by examining the mediating role of psychological stress (PS) on the relationship between fear of infection with COVID-19 (FIC) and EIPD based on affective events theory (AET) and the moderating effect of organizational career support (OCS) on the relationship between PS and EIPD. Based on 865 survey responses provided by mid-level managers from Chinese manufacturing firms and the covariance-based structural equation modeling (SEM) technique using AMOS 25, we identified that FIC has a positive relationship with EIPD while PS can fully mediate the link between FIC and EIPD and OCS weakens the positive relationship between PS and EIPD (that is, in the presence of OCS, EIPD decreases despite the presence of PS among the employees). The findings of our empirical study will theoretically and practically contribute to the pandemic-related existing literature by providing an in-depth understanding of these variables. Furthermore, policymakers can also benefit by boosting their EIP from the outcomes revealed and suggestions provided.

## 1. Introduction

Starting from December 2019, the world has experienced the emergence of a novel disease, the severe acute respiratory syndrome (SARS-CoV-2), widely known as coronavirus disease (COVID-19), declared by the World Health Organization (WHO), which almost altered our way of everyday life [1]. The in-depth effect of this invisible infectious disease brought us to face immense physical and psychological vulnerability. As a matter of fact, COVID-19 disrupted our psychical and mental health, accelerating the mortality rate that caused severe damage in many business sectors. The delayed extension of the evil infection extremely affected the global healthcare system, thus forcing this pandemic time as a global health emergency declared by the WHO [2]. The mysterious COVID-19 forced the entire world population to observe its shocking effects on individuals’ psychological health. Consequently, the pandemic fear has been acknowledged as the most upsetting factor disputing the employees’ work performance resulting from the psychological stress (PS) of fear of being infected across many organizations worldwide [3]. Furthermore, the elevated COVID-19 infectivity or fear of being infected has considerably made individuals anxious about its health repercussions, thereby deteriorating their psychological well-being [4].

Psychological well-being indicates a state of wellness where the individuals can benefit from their abilities and can deal with mental stressors (i.e., depression and anxiety) [5]. The COVID-19 epidemic has inundated public health beyond the level of normal performance. The widespread devastation seems to bring overwhelming effects on global economies for almost all the countries, more or less. Particularly, the psychological impact of the COVID-19 epidemic has challenged the employees who deal with high-stress situations such as longer working hours and fear of being infected by their colleagues and infecting their family members. Consequently, such fears appeared to influence regular job functioning due to superior perceived psychological stress [6]. A number of recent studies have indicated that the deterioration of mental health situation associated with COVID-19 negatively affects an employee’s regular job performance [7,8,9]. In a similar manner, the COVID-19-related psychological concern depicted that augmented depression, anxiety, and stress overly record unstable degrees of psychological vulnerability among the employees [10]. Consistent with this argument, the study proposes that the employees feel the risk of being infected and perceive a kind of PS that, in turn, reduces their creativity and innovation since they feel mentally unsafe. In addition, COVID-19 isolation has disconnected many employees from their families and friends, consequently forcing them to experience defenselessness, nervousness, and anxiety [11]. A study conducted in Pakistan by Imran [12] indicated that healthcare employees during the COVID-19 epidemic enormously experienced the feeling of health-related vulnerability, including depression (26.4%), anxiety (22.6%), and stress (4.4%).

In addition to psychological concerns, COVID-19’s high risk of infectivity and associated psychological distress augmented the feeling of financial stress among employees of many organizations, particularly in the severely affected countries [13]. COVID-19 financially hit various communities, with its extending consequence on millions of individuals who were shocked due to enormous layoffs [14]. The massive turbulence of the pandemic has made many employees lose jobs, creating a feeling of financial stress for them [3]. Such a critical condition led the employees to face economic vulnerability, leading to a sense of exhaustion and anguish. The unforeseen consequences of the pandemic led many organizations to experience global economic despair, increasing the degree of job insecurity of many employees [15]. Possibly, to deal with the present economic subjugation, a successful mechanism is required to be adopted to ensure an instant response to the emerging concerns. Although it is clear that a good amount of research is available on the COVID-19 pandemic, the context of possible predictors of PS and employee innovation performance deficiency (EIPD) during the time of the pandemic, further research needs to be performed, particularly considering the EIPD of mid-level managers [16].

Based on affective events theory (AET), this study is expected to be a pioneering one that assesses the multiple factors such as the level of COVID-19 fear and PS that can negatively affect the innovative performance of mid-level managers. In other words, those factors can increase EIPD among the employees, particularly among the mid-level managers of manufacturing firms. Indeed, COVID-19 is a transmittable disease damaging the workers’ welfare connected to psychological concerns and, ultimately, their innovation performance. AET is based on the notion that people are emotional beings that influence their behavior. Organizational stress, emotions, and sentiments are connected to innovative job performance through AET [17]. As per Martocchio [18], AET argues that emotions are significantly vital in handling workplace activities. According to AET, employee innovation performance, workplace engagement, and job satisfaction are all influenced by the underlying factors and perceptions (i.e., feelings) they encounter at their workplaces. In this regard, affective and subjective supports from the organization are quite essential and required to get the best innovative performance from the employees. EIPD can negatively affect the overall firm performance and competitive competency within the industry. Thus, fear of infection with COVID-19 (FIC) can be positively associated with EIPD and PS, and PS can be positively associated with EIPD.

Manufacturing firms as the focal point of this study, which play the prime elements of the national economy and vital roles in the sustainable development of China’s new era through contributing big shares in the gross domestic product (GDP) as well as global manufacturing output. China became the biggest contributor to the world’s manufacturing sectors and contributed 28.7% of total global manufacturing output in 2019, which is prominently 10% bigger than the United States [19,20]. This sector contributed around USD 4 trillion to the national economy, which is almost 30% of the total economic output of China [19,20]. Following the growth of previous years, industrial sectors in China accounted for 32.6% of the national GDP in 2021 [21]. However, the manufacturing sector is observing a decreasing pattern of contribution to China’s GDP. For example, during the first quarter of 2022, the contribution was reduced to 9355.150 billion Chinese yuan (CNY) from the last quarter of 2021, which was an amount of CNY 37,257.530 billion [22] due to a substantial decrease in production facilities as a result of the continuous COVID-19 outbreak. Furthermore, the total profit of this sector was reduced to 8.3% [23]. As such, as a vital part of the national economy, it is imperative to focus on and improve the employee innovation performance (EIP) of this sector so as to keep its sustained contributions and development.

Miscellaneous features of the mid-level managers’ contributions toward EIP have been documented by a number of researchers [24,25]. The contributions of mid-level managers to an organization’s EIP strategy were studied by another study [26] that was closely connected to innovation and performance [27]. The implications of intellectual capital supported by the organizations are that they are encouraged to take calculated risks without fear of losing their jobs [28]. The innovative procedure in a constituted organization as the precious role and essential characteristics of mid-level managers were recognized first by Felicio et al. [25]. The implications of mid-level managers in escalating and supporting innovations have been discussed by researchers from the international business discipline [29]. Mid-level managers are mainly responsible for operating the various departments of an organization, and they frequently come in contact with the general employees. Therefore, we propose in this paper that mid-level managers can be mainly affected by FIC and PS, thus ultimately reducing EIP or increasing EIPD. However, with proper organizational career support (OCS) from the organization, mid-level managers can reduce EIPD as the guardians of the general employees of an organization.

Although COVID-19 and its subsequent effect on stress, such as job stress [7,30], PS [31,32], mental depression [33,34], psychological safety [9], economic stress [13], decision-making [35], supply chain [36], and so forth are now the common cases and extensive research in academia, its effect on innovation performance deficiency and, more importantly, organizational career support (OCS) as a reactant is still remaining a research lacuna in this domain. Hence in this study, we focus on examining the COVID fear infection and its effect on employee innovation performance (deficiency) (EIP(D)) based on manufacturing firms from China through direct and indirect effects (PS) and reveal how OCS can play as moderating role between PS and EIPD. Hence, we incorporated OCS as the moderator to identify whether it strengthens or weakens the relationship between PS and EIPD. OCS can be explained as the degree to which employees believe that their organization cares about their career growth and values their contribution while offering career growth [37]. OCS is an application of social exchange theory (SET) that explains workplace behavior [38] through the identification of the social behavior of two parties’ interactions. SET offers a cost-benefit analysis to establish the risks and benefits by covering the areas of character, expansion, and the outcome of perceived organizational support (POS) [30,39]. OCS can be intensely explained in the organizational support theory (OST), which identifies that on the basis of the norm of reciprocity, employees make a trade-off between their commitment, efforts, and dedication to their organization for tangible (i.e., pay and other benefits) and non-tangible incentives such as socio-emotional benefits (i.e., esteem, caring, and recognition) and career growth (i.e., promotion) [40]. Based on the above contemporary importance, rationale, and background, we focus on delving and forward the following research questions to make this study more insightful:

RQ1: Does fear of infection with COVID-19 (FIC) reduce employee innovation performance (EIP) or increase employee innovation performance deficiency (EIPD)?

RQ2: What is the indirect effect of psychological stress (PS) from FIC to EIPD?

RQ3: How does organizational career support (OCS) moderate the relationship between PS and EIPD?

We divided this paper into several sections. The first section encapsulates the background and rationale of this study. Section 2 makes an extensive review of the existing literature as well as developed the research hypotheses and conceptual framework. Section 3 eloquently describes the research methodology, whereas Section 4 discusses the research findings and interpreted those findings. Section 5 makes a comparison between our findings with the previous studies. Section 6 points to the implications for theory and practice, while Section 7 highlights the research limitations as well as further scope for investigation. Lastly, Section 8 summarizes the conclusions and recommendations of this study.

## 2. Literature Review, Research Hypotheses, and Theoretical Framework

### 2.1. Employee Innovation Performance Deficiency (EIPD)

In general terms, innovation is the process of generating and implementing new, productive, and useful ideas. Employee innovative performance (EIP) indicates the ability of employee behavioral success intended to achieve the innovative outcome that includes the generation and implementation of novel and inventive ideas by the employees. Even though the term “creativity” is closer to the term “innovation”, creativity is just the beginning point of an innovative process [41,42]. Creativity involves the generation of imaginative new ideas, whereas innovation involves introducing something new, effective, and productive in the market. Literature concerning EIP indicates that all sorts of innovation begin with the primary stage, the generation of ideas, where fresh and constructive ideas can be generated in any area [43]. The second stage is the development of that idea, where the idea innovator builds an alliance of supporters and obtains necessary approvals from his/her colleagues and/or managers. The last stage is the execution of that idea, where it is transformed into useful materialization within a work role or group or inside the entire organization. EIP is a multi-stage process with dissimilar activities, diverse stages of development, and different employee behaviors required at each stage [43].

According to Gilbert, performance deficiency is a disruption that can inhibit individuals from implementing and achieving their highest potential [44] and is divided into continuous/chronic and situational performance deficiencies [45]. Employee performance deficiency is a kind of unsatisfactory work performance when an employee is unable to perform his assigned tasks at an acceptable level within a specific deadline, and it was frequently studied to identify the discrepancies between the required performance and the actual performance [46]. Such performance deficiencies are basically aroused by an employee-related problem such as unsuitability for the assigned job, emotional/external factors, lack of job knowledge, insufficient work facilities, lack of authority, goals, and co-operations, conflicts and counter norms with colleagues and organizational objectives, and so on. In most cases, these performance deficiency-related problems can be corrected by their supervisor, organizational action plan, and support [45]. In this vein, EIPD can be termed as the general lack of creativity and ultimate deficiency in the later stage (i.e., innovation) among the employees at a satisfactory/acceptable level [47]. Such deficiencies in EIP particularly affect the operational performance of R&D departments in manufacturing firms. According to a study conducted by Hanif et al. (2016) in China and Pakistan, Chinese and Pakistani SMEs are particularly influenced by HR generic strategies in promoting knowledge sharing and reducing EIP deficiencies [48]. Therefore, an EIPD, specifically in manufacturing firms, is detrimental to the firm’s overall performance as it can impede the growth and development of the competitive position of a manufacturing firm compared to its rivals.

EIP is essential for achieving different competitive advantages and organizational success. The prevailing literature advocates that employees play a superior role in creating and promoting innovative ideas since they are the source of a range of networks, knowledge, and experiences [49]. Although we observe some debates over how EIP should be defined, the scholars are in consensus that it involves some novel concepts regarding sustainable products/services that can be put into the process [50] rather than just generating the ideas. Business managers should move forward from new idea generation toward refining existing ideas for succession toward the objective and ultimately execution of those novel ideas. Business leaders (or managers) today are under immense pressure to find techniques for increasing EIP due to increased competition, globalization, and the pace of technological revolution. Thus, EIP is now being considered a core competence of the organizations. At present, EIP is no more considered just an inborn quality of some employees [51,52]. Rather, it is more and more being reflected as a dynamic ability to be additionally improved or developed through ample training and experience [53]. Consequently, at present, scholars and practitioners associate overall organizational success with EIP [54]. Organizations are primarily focusing and relying on the innovative performance of their employees, and are predominantly prioritizing discovering the ways to identify and promote EIP as well as discover and reduce the causes that create EIPD [49,55].

### 2.2. Fear of Infection with COVID-19 (FIC)

The COVID-19 pandemic has been commonly observed to create anxiety, fear, and depression in the public. Moreover, the lack of appropriate treatment, medication, and public consciousness of the invisible virus has further increased the feeling of uncertainty [56]. Consequently, the outbreak of the severely infectious COVID-19 pandemic has been observed as a matter of great concern for not just physical health but also psychological well-being. Although the adverse impact of this pandemic on global mental health has so far not been recorded and measured, however, it has been observed that individuals’ levels of fear and anxiety augment, particularly during the appearance of the pandemic and the number of rising cases of infection [57]. It has been observed that confronting uncertain circumstances, particularly when there is a strong risk of being infected and dying, can amplify the individual level of anxiety, stress, and fear. Ultimately, this can lead individuals to engage in defensive behaviors [58].

In another study, it is emphasized that the fear of COVID-19 will have a great impact on mental health, creating severe psychological stress [59]. As a result, the character of the COVID-19 pandemic causes common fear and anxiety [56]. Anxiety and fear, termed as “a state of restlessness or anxiety caused by the expectation of a real or perceived threatening incidence or circumstances” [60], have been identified to be widespread among employees, predominantly the manufacturing sector employees who cannot work from home or online. Furthermore, due to the physical contact with each other, FIC can further increase the fear and anxiety among the employees of the manufacturing sector organizations [61].

### 2.3. Psychological Stress (PS)

Throughout the time of the pandemic, the rising psychological concern accelerated the symptoms of stress and depression among individuals. The increasing physical and mental health-related concerns deeply enhanced the association between the COVID-19 outbreak and mental depression. Such an increasing psychological concern indicated the connection between employee wellness and stress. The unconstructive experiences of employees’ psychological health (i.e., fatigue, stress, and emotional exhaustion) create a kind of fear for their psychological well-being [8]. Consistent with this argument, Schonfeld et al. [62], in their investigative study, indicated that people with elevated stress display feelings of emotional collapse, tiredness, and energy deficiency. The psychological concerns extensively report detachment from workplaces, thereby elevating distress among the workers [63]. Certainly, the recent pandemic has made employees of diverse sectors in bearing the powerful impact of COVID-19-related PS. It particularly scared the manufacturing workers regarding their psychological well-being and communal lives [64]. Thus, employee psychological concerns are required to be emphasized and researched in-depth to cope with the challenging difficulties of the organizations during the epidemic.

### 2.4. Organizational Career Support (OCS)

According to organizational support theory (OST) [65,66,67], employees’ “perceived organizational support” is the one that cares for their well-being and recognizes and values their contributions. The organization itself and managerial and peer-group support are rooted within the social support setting [68]. Particularly, OCS plays an active role in the intrinsic value of psychological concern [68,69]. Hobfoll [69] demonstrate within the conservation of resource (COR) theory that resources such as physical, psychological, societal, or situational are representative of a person’s self-identity. Employees value resources that they receive in the form of support provided by their organization, managers/supervisors, and coworkers [70]. Such perceived sources of support within a healthy and constructive interpersonal association with the team members promote higher perceived psychological safety in the job environment [71,72,73]. In addition, Lee and Chui [74] recommend that OCS and equality in implementing human resource practices augment the psychological well-being of the employees. They established that observing supportive and fair treatment from the organization and managers and adopting an open communication policy with the employees are perceived as career support from the organization.

### 2.5. FIC and EIPD

Studies on epidemics indicate that the prolonged and widespread nature of such pandemics arouses feelings of stress, anxiety, and depression among people [8]. In recent years, COVID-19 has accelerated the fear among the mass people [75], thus escalating the receptiveness of despair among the employees [76] that has substantially reduced their innovative performance or, in other words, increased innovative performance deficiency [77]. Particularly, during the time of the epidemic, some employees of specific job sectors (such as healthcare and manufacturing) have become enormously exhausted due to physical presence and prolonged working hours. The growing FIC considerably resulted in the symptoms of despair among the people, adversely impacting the employees’ psychological health. For example, researchers studying the psychological symptoms connected to the epidemic demonstrate a high occurrence of depression (28%) and anxiety (33%) among Chinese employees [34]. On the other hand, the FIC has considerably influenced employee innovation-based outcomes [8]. Based on the articulation, we argue that increased FIC can lead the mid-level management of the manufacturing sector to exhibit reduced work performance and a severe reduction in their innovative performance dynamics [78].

The heightened fear of COVID-19 demonstrated a significant decline in EIP due to the rising psychological troubles [79]. A strong psychological state influences the employees’ work performance. Certainly, the recent pandemic has accelerated many psychological issues, causing many employees to be in difficult situations when performing their jobs [80]. According to psychologists, fear requires a defensive response [81], and when the fear is uncontrollable, it turns into a feeling of anxiety. In recent years, the increased FIC has shown an extreme emotional influence on employees’ mental health, fundamentally making them work with an anxiety disorder [82]. Such an adverse psychological state of mind is not conducive to regular job performance and, ultimately, decreases EIP [82]. In addition, the COVID-19 pandemic has boosted the stress symptoms among individuals [8]. Stress is a protective reaction that requires physical, emotional, and psychological adjustment [83]. Different people respond to stress in a different manner based on emotional, physical, and psychological factors.

FIC has drastically impacted employees’ psychological health, forcing them to encounter significant distress. The amplified emotional fatigue, loss of energy, and exhaustion adversely affected employees [84], thus significantly reducing their ability to think, act, and create. Particularly, the FIC enormously uncovered the R&D employees’ as well as mid-level managers’ creativity and innovative performance due to the vulnerable effects of psychological stress [85]. The COVID-19 situation led many employees to react to stressful situations, thus damagingly impacting their EIP [77]. A recent study shows that 112 million individuals reported the symptoms of extreme stress in China [32] that has drastically reduced their creativity and innovative performance or, in other words, increased their EIPD. According to AET, there are numerous factors that can adversely affect the EIP [17]. Based on such theoretical ground, we propose that the level of COVID-19 fear (i.e., FIC) can have a positive impact on the rising EIPD of employees. We propose that those negative psychological factors can increase EIPD among the employees, particularly among the mid-level managers of manufacturing firms. Indeed, COVID-19 is a transmittable disease that can certainly damage the employees’ welfare with psychological concerns and, eventually, increases their EIPD. AET is based on the notion that people are emotional beings that influence their behavior. The theory states that organizational stress, emotions, and sentiments are connected to innovative job performance [8,49]. Consequently, based on the arguments, we propose to test the following hypothesis:

**H1.**  *FIC has a positive relationship with EIPD.*

### 2.6. FIC, PS, and EIPD

At the time of the pandemic, the growing PS accelerated the symptoms of despair among the employees. Such an increasing psychological concern clearly indicates the underlying connection between FIC and employee PS [8]. The negative experience on employees’ mental health (i.e., tiredness and emotional exhaustion) made the employees worried about their mental health. A study highlights that individuals with elevated depression demonstrate feelings of emotional collapse, exhaustion, and, ultimately, loss of energy that affects their innovative performance [62]. The rising psychological concerns broadly account for the lack of EIP [8,77] and, finally, detachment from work [30]. Undeniably, the current pandemic forced the manufacturing sector employees to bear the influential effect of the COVID-19-related FIC and PS. It acutely scared the manufacturing workers about their psychological welfare and community life [64]. Thus, employees’ psychological concerns need to be emphasized and studied, coping with the challenging requirements of the workplace during emergencies such as the COVID-19 outbreak.

Recently, ensuring the employees’ psychological well-being has turned into a major area of concern for behavioral researchers [8]. Identifying and acknowledging the central point of defending employees’ welfare is significantly required to ensure to get the best out of them in the situations such as the COVID-19 outbreak. Fulfilling the psychological requirements can make the employees increase their work motivation and regain their missing energy. Particularly, research showed that psychological health issues reduced individual work performance and raised the chances of more mistakes [86], adversely affecting task performance and raising the symptoms of distress and mental illness among individuals [86]. Noticeably, during the recent COVID-19 outbreak, the psychological damage (i.e., fear and depression) has led to a decline in the employees’ overall health conditions [31]. In addition, the amplified psychological concerns during this epidemic have made employees come across undue mental stress, thus affecting their EIP [87].

Noticeably, during the COVID-19 epoch, the mid-level and lower-level manufacturing sector employees have become susceptible to mental issues such as fear of being infected and anxiety as they have to be physically present at the workplace and cannot work online like service sector employees. In line with this argument, recent research involving 97,333 employees from 21 countries has demonstrated an elevated existence of anxiety symptoms (22.1%) among healthcare workers [33]. During COVID-19, the likelihood of infecting family members and friends troubled the employees [88]. Psychological well-being contributes to employees’ contentment, pleasures, and individual development [89]. Few studies identified that indecisive circumstances such as the pandemic make the employees lose control over their personal lives as well as jobs [90]. The extensive spread of the disease made the employees stressed about its high risk of infectivity [91], reducing their innovative capability and job performance. On the other hand, AET, which has been popularly contributing to the role of occurrence-based emotions on job performance, states that negative emotions and stress can reduce innovative performance [8,17,49]. Based on the grounds of AET as well as those study outcomes, we argue that during the COVID-19 outbreak, a dominant proportion of mid-level and lower-level employees are experiencing FIC and symptoms of stress, which consequently reduces their EIP or increases their EIPD [61]. Thus, based on the prior literature, we propose to test the following hypotheses:

**H2.**  *FIC has a positive relationship with PS.*

**H3.**  *PS has a positive relationship with EIPD.*

**H4.**  *The relationship between FIC and EIPD can be mediated by PS.*

### 2.7. PS, OCS, and EIPD

The existing literature indicates that career support by the organization can positively influence job performance and employee satisfaction through positive and vibrant reciprocity [14,79]. As a result of perceived organizational career support (OCS), employees become motivated and exhibit their superior innovation performance in response to the organization’s constructive atmosphere, reward policies, and supervisory treatments, even during times of high work pressure and mental stress [92]. Further, an effective OCS policy can reduce the employees’ perceived PS and feel them empowered, committed, and contributive toward innovative performance [93]. Alshaabani et al. (2021) identified that perceived organizational support, such as career support, is positively related to organizational citizenship behavior that eventually induces employee performance [94]. Rhoades and Eisenberger [67] proposed some organizational elements that can contribute to employees’ perceived OCS, such as justice and fairness in reward and promotional policies, managerial support, and job conditions (i.e., job autonomy, role stressors, and training) [9]. Managerial/supervisory support is connected to OCS since employees consider them as the agents or representatives who act on behalf of the organization [27]. Such perceived support has also been empirically revealed to amplify employees’ career satisfaction [95]. An employee who receives organizational career assistance will feel obliged to return the favor (i.e., reciprocity) toward the organization [96]. OCS also helps employees satisfy socio-emotional requirements such as recognition, association, and esteem, reducing job stress, and lifting employee wellness and career fulfillment [97]. Empirical studies have shown that perceived supervisor support leads to positive OCS, influencing employee career satisfaction [98].

Antecedents of career satisfaction are one of the contemporary frequently studied domains of organizational behavior [99]. Organizational concentration on developing career-related skills can successfully raise employees’ sense of feeling in their job environment [100]. OCS is one of the significant indicators of employees’ career satisfaction. It encourages the socialization procedure within the organization, which in turn promotes the acquisition of pro-social values and institutionalized civic service [101]. When the OCS and interior sustainability permit the employees to satisfy their proficiency and independence requirements, conductive organizational attitudes are developed [102]. An earlier study established that OCS predicts job crafting, creating satisfaction among employees within the organization [103]. In turn, OCS is positively correlated with superior work engagement, higher job commitment, and, subsequently, superior EIP [104]. This study intends to extend the current literature by examining the moderating role of OCS in the relationship between PS and EIPD of the Chinese mid-level manufacturing sector employees. Thus, we propose to test the following hypothesis:

**H5.**  *OCS can weaken the positive relationship between PS and EIPD; that is, in the presence of OCS, EIPD decreases despite having PS among the employees.*

### 2.8. Conceptual Model

**Figure 1:**
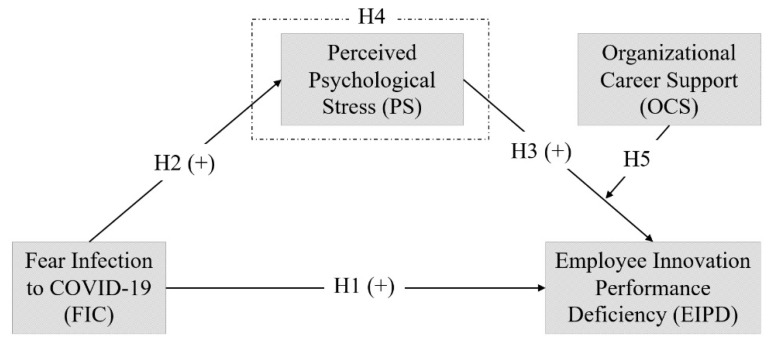
The proposed conceptual framework for the hypothesized relationships.

Our study has one dependent (FIC), one independent (EIPD), one mediator (PS), and one moderator (OCS) variable. The conceptual framework of our study is depicted in Figure 1:

## 3. Research Method

### 3.1. Survey Design and Data Collection Process

This cross-sectional research collected data during the period of COVID-19 (July 2021 to March 2022) inside mainland China. Survey respondents were drawn from industry-driven and major cities in China such as Chuzhou, Shenzhen, Guangzhou, Huizhou, Ningbo, Wenzhou, Shanghai, Chongqing, Chengdu, Fuzhou, Wuxi, Xiamen, Zhongshan, and so on. These cities contain many potential mid-level managers for our research and are vulnerable to pandemics due to their population and manufacturing facilities, consequently having acute FIC. Thus, respondents from these regions provide diverse backgrounds for sampling with economic, industry categories and size, income and expenditures levels, cultural, technological, and psychological aspects.

We utilized the purposive sampling technique to choose the participants to collect preferred primary data from the specific groups, industries, and levels of employees, who were able to provide the required data for this study with maximum variation but homogenous sampling [105]. This technique also allowed us to meet the specific criteria for the typical cases by purposively choosing the potential participants [106] who possess relevant experience and information regarding our research variables such as FIC, PS, OCS, and EIPD. This purposive sampling selection guided us to avoid non-relevant participants and prevent 80–100% biasing [107] rather than using random/automatic sampling to get a large number of answers from fewer rational responses.

The survey was monitored and administered by four research teams, including one researcher and three trained research assistants from the university level in China (i.e., undergraduate students). A pilot survey was conducted with a small number of participants to ensure and validate the grammar, rationality, readability, and dimension issues of the questionnaires so as to revise them before starting the formal and final version of data collection from respondents. The survey was conducted through email using the electronic version of questionnaires to maintain prevention policies of COVID-19 as well as ease us to cover all targeted cities for our research. A detailed electronic manual covering rules, processes, survey items, and aims for the research was provided along with the questionnaires to ease the respondents’ complexity of the answering process. Furthermore, respondents were free to contact our monitoring team to avoid any misunderstandings in filling out the questionnaires. Before beginning the responses, survey participants were asked to fill out a protection form concerning their willingness to voluntarily participate as the responder. They were also well assured about the anonymity and confidential policy about their personal information. A small gift token was provided upon successful completion of the filling questionnaires, which aimed to encourage them to properly and accurately fill the questionnaires. These efforts, on the one hand, encourage them to freely, anonymously, and completely answer the questionnaires, which guided them to reduce social desirability related to common method bias in the data collection process [108]. On the other hand, it helped to reduce the non-response rate and control the non-response bias with a minimum rate [107,109].

In the actual survey, the final version of the questionnaires was sent to 1020 targeted respondents in our purposive sampling groups (i.e., mid-level managers in manufacturing industries from major cities of China). After willingly participating and filling out the questionnaires, we received 922 returned answers from the participants with a response rate of 90.4%. However, incomplete (e.g., responses for some items were missing) and incorrect (e.g., misinterpreting the questions, responding to all items to the same scale, not following proper instructions, and so on) answers were discarded after carefully reviewing the datasets. Hence, in the final stage, 865 useable responses were kept as the valid final datasets with an accumulated response rate of 84.8% and listed in detail with their profile information in Table 1. The remaining non-response rate of 15.20% was considered to be the acceptable range of non-response bias in our study [109].

In Table 1, data from respondents include gender, age, education level, experience, and so forth with their corresponding range, count, and percentage. For example, in gender profile, male (60.12%) has a higher proportion than female (39.88%); the majority of mid-level managers’ age is from 36 to 45 and have 11–20 years of work experience with a 49.48% proportion; majorities are married (72.60%), and top proportion of industry categories belong to machinery and vehicles (20.58%).

### 3.2. Measurement Technique and Tool

In accordance with the relevant literature, the questionnaire was adapted by using the survey instrument. For example, we adopted the scale developed by Soraci et al. [82] for measuring the FIC using 5 items. Such questionnaires were aimed to get respondents’ profile information as well as their responses to the survey. Our survey comprised a total of 4 constructs/variables, and each construct comprised 4–5 items with a total of 19 items to investigate the relationship between the variables. Each item adopted a 7-point Likert scale, the widely validated and tested scale in existing research, where scale 1 represented “strongly disagree” and scale 7 represented “strongly agree”. Construct, content, and face validity were assessed carefully before collecting data from respondents [110]. To do so, 4 scholars, 10 managers from manufacturing firms, and 5 experts from psychology and innovation were invited for questionnaire evaluation. This process further guided us to adapt/modify the questionnaire/measures for constructs from the relevant literature and attain our objectives within its context by maintaining the validities. Finally, each questionnaire was delivered to respondents in bilingual mode (i.e., Chinese and English) and performed back-translation technique to increase the reliability of the contents. Table 2 displays the variables, items, and their relevant coding used for this survey.

## 4. Empirical Analysis and Interpretation

### 4.1. Descriptive Statistics and Correlations

Correlations among the constructs and their descriptive statistics (i.e., mean and standard deviation) are reported in Table 3. From the correlation matrix, it is observed that most of the correlation coefficients among the constructs are positively significant (ranging from 0.089 to 0.557) with a maximum *p*-value < 0.01 and are consistent as per our assumption, explained through the hypotheses. Reported mean and standard deviation (SD) values also show less variance, and all SDs are below the mean values.

### 4.2. Testing Reliability, Validity, Method Biases, and Model Fitness

Assessing reliability and validity test of the model were verified through several standard measurement scales criteria, which are most widely used in the existing literature. All the reliability and validity testing were conducted through SPSS and its companion software AMOS (Windows Version 25.0), released by IBM, Armonk, NY, USA. The results are reported in Table 4 to compare their corresponding standard measurement scales. In the reliability and validity testing results, we have reported factor loadings (FLs), Cronbach alpha (CA), composite reliability (CR), average variance extracted (AVE), maximum shared variance (MSV), and average shared variance (ASV) for each item of the construct.

In this study, first, the reliability test was verified by reporting FL and CA. As per the reported results, all the FLs ranged from 0.712 to 0.929, displaying all FL > 0.50, which is the minimum FL threshold scale [111,112] to ensure better confidence recommended by Hair et al. (1998) and Field (2009). For the CA results, we found that the minimum CA value was 0.798, which also conformed to and surpassed the threshold value of 0.60 [110] recommended by Nunnally and Berstein (1994).

Second, we conducted the validity test by comparing the standard scales of convergent and discriminant validity with our reported results. For the convergent validity test [113], we found that our reported results are CR > 0.70, AVE > 0.50, and CR > AVE for all constructs. For the discriminant validity test, we observed that for all constructs: AVE > MSV and AVE > ASV. Thus, both the convergent and discriminant validity was confirmed by maintaining standard measurement scales, which shows that our data and scales are valid and correct [114].

Third, we examined common method variance (CMV) using the common latent factor (CLF) to check and ensure the variance for all the variables used in our model. CMV refers to the number of spurious covariances shared among the variables due to the common method applied in collecting data [115,116]. Such method biases can be problematic due the actual circumstance under investigation becomes difficult to differentiate from measurement artifacts [115]. All reported estimates of CMV effects as per Table 4 are less than 0.2, which asserts that there is no issue for common method bias for our analysis [108].

Finally, we examined the model fitness through confirmatory factor analysis (CFA) to check whether, under similar conditions, the model was able to reproduce the linkage with other data. The model fitness indices are listed in Table 5 and showed that all indices such as Chi-squared (χ^2^)/degrees of freedom (df), comparative fit index (CFI), root mean square residual (RMR), the goodness of fit index (GFI), adjusted goodness of fit index (AGFI), Tucker–Lewis index (TLI), root mean square error of approximation (RMSEA), and standardized root mean square residual (SRMR) of direct and indirect models belong to the acceptable standard range. For example, for the direct effect model, χ^2^/df = 3.221; AGFI, CFI, GFI, and TLI > 0.925; RMR, RMSEA, and SRMR < 0.060, and for indirect effect model, χ^2^/df = 2.922; AGFI, CFI, GFI, and TLI > 0.904; RMR, RMSEA, and SRMR < 0.063.

### 4.3. Hypotheses Test

Both the structural equation modeling (SEM) using AMOS (Windows version 25.0) and PROCESS macro version 4.1 [117] installed in SPSS, Windows version 25.0 (IBM, Armonk, NY, USA) were used to test the hypotheses for direct and indirect effects. We have assessed two models to test all effects in the path using the linear moderated mediation approach by Hayes (2015) [118] and report in Table 6. First, we assessed the direct link between independent (FIC) and dependent (EIPD) variables. Second, we assessed the mediating and moderated mediation linkage of PS and OCS on PS to EIPD.

#### 4.3.1. Testing Direct Effect Path

The direct path between FIC and EIPD was assessed through total effect as presented in Equation (1). As shown in Table 5, all the goodness of fit indices were within the standard accepted range. For the results from the direct effect using Equation (1,) we found a positive significant association between FIC and EIPD (β = 0.211, S.E = 0.068, CI = [0.177, 0.259], *p* < 0.001). Thus, H1 is supported.

#### 4.3.2. Testing Indirect Effect Path

In the indirect path testing, we assessed PS as the mediator variable between FIC and EIPD through the bias-corrected bootstrapping at 5000 resamplings and a 95% confidence interval (CI) level. As per Table 5, the goodness of fit indices of all constructs were within the accepted range. In testing the indirect effect, first, we examined FIC to PS using Equation (2) and found the significant positive relation (β = 0.3426, S.E = 0.019, CI = [0.305, 0.380], *p* < 0.001) between them. Thus, H2 is supported in this study. Second, we examined the conditional indirect effects using Equation (3) to test the path effect of PS to EIPD and OCS as a moderator in a similar path. We found a significant positive relationship between PS and EIPD (β = 0.552, S.E = 0.030, CI = [0.558, 0.712], *p* < 0.001). Thus, H3 is supported. In this stage, we further reassessed total, direct, and indirect effects and found no significant relationship between FIC and EIPD as the direct effect. Hence, H4 is supported as the full mediation in the path. We tested the conditional indirect effect as moderated mediation using the second stage moderated mediation approach and model 14 described by Hayes (2015) [118].

*Y = i_Y_ + cX + e_Y_* (1)

*M = i_Med_ + a_1_X + e_Med_* (2)

*Y = i_Y_ + c′X + b_1_M + b_2_W + b_3_MW + e_Y_* (3)

*θ (indirect effect) = a_1_(b_1_ + b_3_W)* (4)

where *c* represents the total effect. *c*′ represents the direct effect. *X* represents fear of infection with COVID-19 (FIC). *M* represents psychological stress (PS). *W* represents organizational career support (OCS). *Y* represents employee innovation performance deficiency (EIPD). *θ* represents the conditional indirect effect.

According to the second stage moderated mediation model, the indirect effect is functioned as *a_1_* (*b_1_ + b_3_W),* which is the linear function of the moderator (OCS) and is displayed here in Equation (4). Based on Equation (4), the indirect effect of FIC on EIPD is the product of effects calculated in Equation (2) and conditional indirect effects calculated for PS on EIPD in Equation (3); thus, the indirect effect of the moderated mediation of FIC on EIPD can be calculated as *a_1_b_3_.*

**Figure 2:**
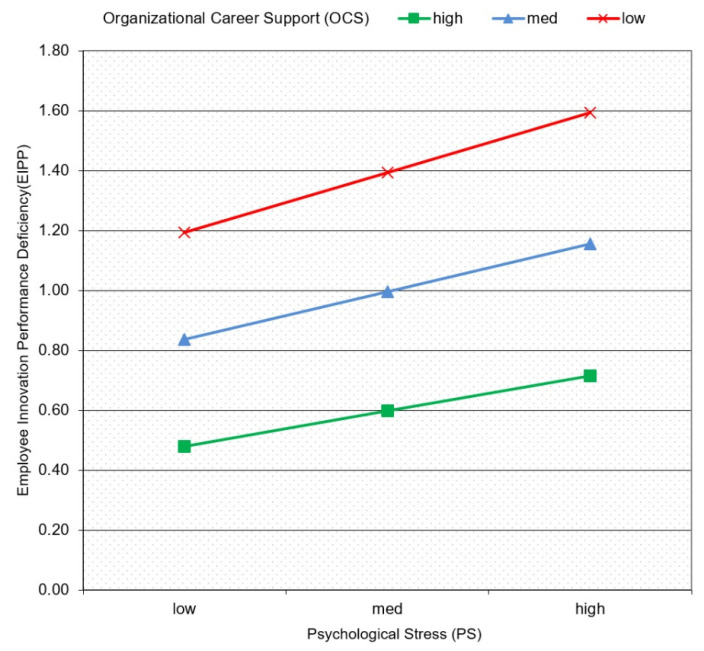
The moderating effects of OCS on PS and EIPD.

Finally, we reported different levels of moderation effects (i.e., above one SD from mean as OCS = high, SD at mean as OCS = medium, and below one SD from mean as OCS = low) to test moderated mediation effect of FIC on EIPD through PS. Results also found a significant decreasing effect of PS on EIPD in the presence of OCS. To understand the moderating effect more visually, we graphed the moderating effects in Figure 2. According to the moderation effect in Figure 2, it is clear that OCS negatively moderates the relationship between PS and EIPD; that is to say, the effect of PS on EIPD is decreasing by increasing the OCS. Based on the above, H5 is supported.

## 5. Discussion and Findings

This study identified that FIC has a significant positive relationship with EIPD or, in other words, FIC has a significant negative relationship with EIP. Such an outcome is consistent and similar to the previous findings reported by Sarfraz et al. [8]. On the other hand, numerous authors such as Shah et al. [7]; Lee [9]; Lei et al. [10]; Kang et al. [11]; Thayer and Gildner [13]; Tu et al. [14]; Abbas et al. [15] argued that the COVID-19 had accelerated fear among people, thereby escalating the receptiveness of despair among the employees that have substantially reduced their innovative performance [77]. The same factor was found to have a significant positive relationship with PS. This outcome is also supported by the previous studies conducted by Torales et al. [59], Pappa et al. [61], and Presti et al. [64], who reported that the rising physical and mental health-related concerns deeply enhanced the association between the COVID-19 outbreak and mental depression. Such an increasing psychological concern explains the association between employee wellness and stress. The unconstructive experiences of employees’ psychological health (such as fatigue, stress, and emotional exhaustion) create a kind of fear for their psychological well-being [8].

On the other hand, we revealed that PS has a significant positive relationship with EIPD, consistent with the findings of Montani and Staglianò [77] and Clercq et al. [87], who identified that rising stress due to the fear of COVID-19 infection could considerably reduce EIP. We also revealed that PS can fully mediate the relationship between FIC and EIPD. This result is also supported by a number of scholars such as Sarfraz et al. [8], Hennekam et al. [86], and Clercq et al. [87]. Finally, we identified that OCS weakens the positive relationship between PS and EIPD (that is, in the presence of OCS, EIPD decreases despite having PS among the employees). Such an outcome is largely supported by Oubibi et al. [92], Qiu et al. [96], and Crucke et al. [98], who argued that in any situation, organizational support such as career support or psychological counseling could be of great benefit to reduce employee stress and increase innovative performance. However, in times of pandemic-like situations, such supports are more necessary to hold employee morale and keep them enthusiastic about their job and organization [70].

## 6. Theoretical Implications and Practice

### 6.1. Theoretical Implications

The continuous wave of the COVID-19 pandemic revolving from place to place and its multidimensional effects hinder the of start new initiatives through creativity and innovation. Particularly in the biggest populated countries such as China, there are obvious fears of being infected that can increase psychological stress and ultimately decrease EIP. However, so far, issues such as FIC, EIPD, and OCS have been largely unexplored. Therefore, we expect that this empirical investigation will have significant implications for academicians and practitioners since it highlights the impact of fear of being infected in a pandemic situation in a biggest populated country. As expected, fear of infection has an increasing psychological impact on the individuals, and such an impact can enhance PS that can reduce the innovation performance of the employees working in this pandemic. On the other hand, it was discovered that OCS can reduce the impact of PS and reduce EIPD in the presence of PS. We believe that more and more empirical evidence will be unveiled regarding the mental health and innovative performance of the employees in the upcoming times of the pandemic. For the guidance of further investigation, it is expected that the findings of this study can contribute to further empirical examination as this pandemic is rotating continuously.

The findings of this empirical study can bring immense theoretical guidelines for the academicians for further investigation of mental health and performance issues during pandemic situations. Although there are several papers that have focused on employees’ fear of COVID-19 and psychological stress, this is the first study that linked the fear of infection, PS, OCS, and EIPD in one frame. Thus, we firmly believe that our empirical findings will enrich the academia and pandemic-related literature.

### 6.2. Practical Implication

In addition to the theoretical contribution, we expect our findings will be beneficial for the organizational policymakers and top-level managers in making decisional policies that can curb fear and stress among the employees. We identified that OCS is a vital tool for employee retention and motivation that can reduce their PS and engage them in the innovative process. Once employees are free from their future worries and can come out of fear and stress, they can concentrate on their creative and innovative processes in the organization that benefit the organization in the long term.

Therefore, this paper can guide managers in their decision-making process regarding how to support their competent employees while making them engaged with innovation. Such support should be included in the policy-making process and can be reflected in the HR policies not only in pandemic-like situations but also in similar circumstances (i.e., national/global economic crisis, war). Let the employees feel that they can grow and flourish if they continue with their organization, and such support will empower them physically and mentally.

## 7. Limitations and Further Scope

We admit that this study possessed several limitations, and those limitations should be highlighted. First of all, we conducted our investigation based on one county and particular culture. A cross-country examination might have provided some more interesting and insightful outcomes. Some of the findings of our study may not be consistent with other cultures or economies as there are differences in personal needs and cultural settings. Second, this study was drawn from a particular level (i.e., mid-level) of respondents, which may limit and affect the generalization of our findings regardless of similar/different characteristics of other groups of respondents (i.e., field level, workers, managers, CEO, owner, and so on). Thus, further study can investigate based on varying/other levels of respondents for this research concept to compare our findings. Third, we used a cross-sectional survey, which is often associated with common method bias. Thus, this study makes it possible to draw further study (i.e., a longitudinal study) for sequential comparison. Finally, there is a slight uncertainty that people are not constantly the best judges of their abilities and limitations. We expect that further research initiatives will consider these limitations in their attempts.

## 8. Conclusions and Recommendations

In our study, we strived to empirically explain the effects of fear of being infected, leading to increased PS and EIPD. Different emotional consequences were targeted in this study to understand the multidimensional effects and vulnerabilities induced by COVID-19, such as COVID-19 fear and stress. This study possibly offers a larger significance by combining the necessary elements required for a clear picture of the overwhelming effects of the pandemic. Our findings particularly demonstrate that this universal picture of the exclusive disaster pessimistically influenced individuals’ psychological health and innovative performance. This disastrous extensive disease provoked anxiety and fear in individuals, thus, declining the performance of the manufacturing sector workforce.

However, we demonstrated that despite such fear and stress, if the employees get proper career support from their respective organizations, they tend to have increased innovative performance and organizational proactive behavior. Therefore, we recommend having an effective career support policy for all organizations. Regarding the COVID-19 intensity, the study indicates the need for extensive career support services embedded in organizational HR policies to ensure the safety and growth potentials of the competent workforce. Therefore, the impact of COVID-19 on perceived fear, psychological concerns, and innovative performance deficiency fundamentally requires designing an effective organizational strategy for achieving better employee outcomes.

## Figures and Tables

**Table 1:** Profile information of the survey participants.

Description	Range	Count	Percentage
Gender	Male	520	60.12
	Female	345	39.88
	Total (*n*)	865	100
Age (Years)	<31	120	13.87
	31–35	210	24.28
	36–45	355	41.04
	>45	180	20.81
	Total (*n*)	865	100
Educational level	College and diplomas	251	29.02
	Bachelors	420	48.55
	≥Postgraduates	194	22.43
	Total (*n*)	865	100
Job experience (Years)	<11	168	19.42
	11–20	428	49.48
	>20	269	31.10
	Total (*n*)	865	100
Marital status	Married	628	72.60
	Unmarried	237	27.40
	Total (*n*)	865	100
Industry category	Machinery and vehicles	178	20.58
	Electronics	158	18.27
	Home appliances	126	14.57
	Textiles	116	13.41
	Packaging	102	11.79
	Chemicals	99	11.45
	Toys	86	9.94
	Total (*n*)	865	100

Source: survey instrument.

**Table 2:** Constructs and survey items with literature sources.

Variables	Survey Items	Coding	References
Fear of Infection with COVID-19 (FIC)	I am afraid of being infected with COVID-19	FIC1	Soraci et al. [82]
	I am afraid that my facility members can be affected by me.	FIC2	
	Since I came to contact with other people, I am really worried I am carrying the virus.	FIC3	
	I am psychologically stressed and emotionally exhausted due to the fear of getting infected.	FIC4	
	I can see people are getting infected, isolated, and losing their jobs due to unstable work and the economy.	FIC5	
Psychological Stress (PS)	A sound mental health is quite essential for my overall performance in the workplace.	PS1	Schonfeld et al. [62]; Nasharudin et al. [63]
	Fear of COVID-19 infection is making me mentally weak, increasing my psychological stress.	PS2	
	I always think about myself and my family during this epidemic, increasing my psychological stress.	PS3	
	During the pandemic, it becomes tough to physically communicate with my teams, reducing sympathy and relationship and increasing my psychological stress.	PS4	
	During the pandemic, I am often afraid about my work performance due to the distance and unable to have close discussions with my teams.	PS5	
Employee Innovation Performance Deficiency (EIPD)	Due to psychological stress in the pandemic, I am worried about my job, impacting my creativity and innovative idea.	EIPD1	Baer [42]; Alghamdi [43];
	Due to the pandemic and stress, often, I am unable to exhibit my excellence, impacting my innovative performance negatively.	EIPD2	
	Due to mental stress, I often lack work spirit, which severely constrained me from thinking of something new.	EIPD3	
	Although I have innovative ideas, but due to mental stress and lack of close discussion, often not interested to share my ideas with others.	EIPD4	
	A proper career support from an organization can improve my innovation performance during COVID-19 pandemic.	EIPD5	
Organizational Career Support (OCS)	Organizational career support is essential for the employee’s overall performance.	OCS1	Kurtessis et al. [65]; Rhoades and Eisenberger [67]; Eisenberger et al. [66]; Lee and Chui [74]
	Organizational career support can reduce mental stress during the pandemic.	OCS2	
	I feel motivated and empowered if I receive career support from my organizations and department.	OCS3	
	Effective organizational career support can reduce PS, job stress, and turnover intention, which increases employee innovation performance during the pandemic.	OCS4	

Note: FIC: fear of infection with COVID-19; PS: psychological stress; OCS: organizational career support; EIPD: employee innovation performance deficiency. Source: Literature survey.

**Table 3:** Correlations and descriptive statistics (mean, and standard deviation) (*n* = 865).

Constructs	FIC	PS	OCS	EIPD
Fear Infection with COVID-19 (FIC)	1			
Psychological Stress (PS)	0.489 ***	1		
Organizational Career Support (OCS)	0.089 **	0.271 ***	1	
Employee Innovation Performance Deficiency (EIPD)	0.205 ***	0.557 ***	0.209 ***	1
Mean Value	4.80	4.73	4.30	4.69
Standard Deviation	0.82	0.78	0.65	0.73

Note: ** *p* < 0.01; *** *p* < 0.001. Source: descriptive statistics (SPSS 25).

**Table 4:** The reliability and validity analysis of the measured variables.

Constructs	Coding	FL	CMV Effects	CA	CR	AVE	MSV	ASV
Fear of Infection with COVID-19 (FIC)	FIC1	0.842	0.095	0.798	0.890	0.617	0.239	0.068
	FIC2	0.811	−0.011					
	FIC3	0.712	0.063					
	FIC4	0.799	0.005					
	FIC5	0.763	−0.078					
Psychological Stress (PS)	PS1	0.929	−0.104	0.887	0.937	0.748	0.310	0.193
	PS2	0.873	0.097					
	PS3	0.841	0.065					
	PS4	0.887	−0.043					
	PS5	0.787	−0.076					
Organizational Career Support (OCS)	OCS1	0.912	0.005	0.842	0.894	0.680	0.073	0.036
	OCS2	0.835	−0.088					
	OCS3	0.729	0.054					
	OCS4	0.813	0.099					
Employee Innovation Performance Deficiency (EIPD)	EIPD1	0.878	−0.072	0.819	0.898	0.639	0.310	0.105
	EIPD2	0.729	−0.035					
	EIPD3	0.811	0.021					
	EIPD4	0.813	0.028					
	EIPD5	0.758	−0.055					

Note: FL: factor loadings; CMV: common method variance; CA: Cronbach alpha; CR: composite reliability; AVE: average variance extracted; MSV: maximum shared variance; ASV: average shared variance; FIC: fear of infection with COVID-19; PS: psychological stress; OCS: organizational career support; EIPD: employee innovation performance deficiency. Source: SPSS 25 and SEM (AMOS 25).

**Table 5:** The model fit indices and their acceptable thresholds.

Fit Indices	Direct Effect	Indirect Effect	Acceptance Threshold	References
Chi-squared/DF	2.922	3.221	<5.0	Marsh and Hocevar [63], Hooper et al. [64]
CFI	0.963	0.947	>0.90	Hu and Bentler [65], Hooper et al. [64]
RMR	0.059	0.062	<0.08	Hu and Bentler [66], Hooper et al. [64]
GFI	0.958	0.933	>0.90	Joreskog and Sorbom [67], Hooper et al. [64]
AGFI	0.926	0.905	>0.85	Anderson and Gerbig [68], Hooper et al. [64]
RMSEA	0.052	0.057	<0.08	Browne and Cudeck [69], Feinian et al. [70]
SRMR	0.051	0.055	<0.08	Browne and Cudeck [69], Feinian et al. [70]

Note: AGFI: adjusted goodness of fit index; CFI: comparative fit index; DF: degrees of freedom; GFI: goodness of fit index; RMR: root mean square residual; RMSEA: root mean square error of approximation; SRMR: standardized root mean square residual. Source: SPSS 25 and SEM (AMOS 25).

**Table 6:** Results of direct and indirect effects.

Hypotheses	Path/Items	Coefficient (β)	BC Bootstrapped Estimates 95% CI	S.E.	Comment
H1	FIC → EIPD (*c = total effect*)	0.211 ***	0.177, 0.259	0.068	Supported
H2	FIC → PS (*a_1_*)	0.3426 ***	0.305, 0.380	0.019	Supported
H3	PS → EIPD (*b_1_*)	0.552 ***	0.558, 0.712	0.030	Supported
	FIC → PS → EIPD	−0.028 **	−0.084, 0.051	0.039	
H4	_OCS = low:_ FIC → PS → EIPD	0.227 ***	0.186, 0.272	0.022	Supported
	_OCS = Medium:_ FIC → PS → EIPD	0.189 ***	0.158, 0.226	0.017	
	_OCS = High:_ FIC → PS → EIPD	0.151 ***	0.135, 0.189	0.022	

Note: ** *p* < 0.01; *** *p* < 0.001; FIC: fear of infection with COVID-19; PS: psychological stress; OCS: organizational career support; EIPD: employee innovation performance deficiency.
